# Laboratory Diagnosis of Invasive Aspergillosis: From Diagnosis to Prediction of Outcome

**DOI:** 10.1155/2013/459405

**Published:** 2013-01-14

**Authors:** Richard C. Barton

**Affiliations:** Mycology Reference Centre, Department of Microbiology, Leeds Teaching Hospitals Trust, Leeds LS1 3EX, UK

## Abstract

Invasive aspergillosis (IA), an infection caused by fungi in the genus *Aspergillus*, is seen in patients with immunological deficits, particularly acute leukaemia and stem cell transplantation, and has been associated with high rates of mortality in previous years. Diagnosing IA has long been problematic owing to the inability to culture the main causal agent *A. fumigatus* from blood. Microscopic examination and culture of respiratory tract specimens have lacked sensitivity, and biopsy tissue for histopathological examination is rarely obtainable. Thus, for many years there has been a great interest in nonculture-based techniques such as the detection of galactomannan, **β**-D-glucan, and DNA by PCR-based methods. Recent meta-analyses suggest that these approaches have broadly similar performance parameters in terms of sensitivity and specificity to diagnose IA. Improvements have been made in our understanding of the limitations of antigen assays and the standardisation of PCR-based DNA detection. Thus, in more recent years, the debate has focussed on how these assays can be incorporated into diagnostic strategies to maximise improvements in outcome whilst limiting unnecessary use of antifungal therapy. Furthermore, there is a current interest in applying these tests to monitor the effectiveness of therapy after diagnosis and predict clinical outcomes. The search for improved markers for the early and sensitive diagnosis of IA continues to be a challenge.

## 1. Introduction

**Figure 1:**
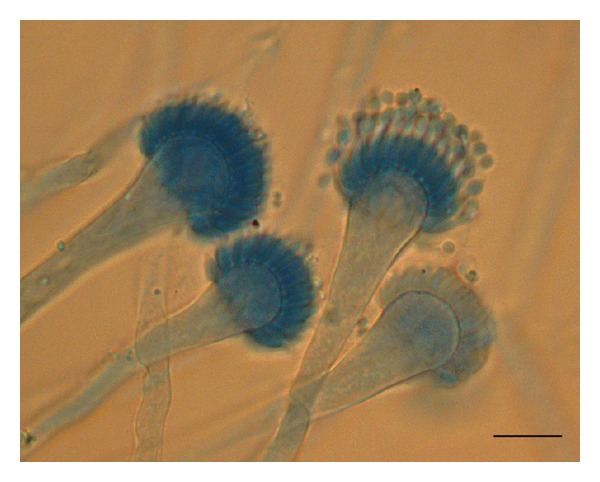
*A. fumigatus *(bar is 10 um).

Aspergillosis, which can be defined as an infection or disease caused by fungi in the genus *Aspergillus* constitutes a wide range of disease entities that form a continuum from allergic reactions to disseminated invasive disease in immunocompromised patients [1]. The specific term invasive aspergillosis (IA), often defined in relation to the primary affected organ as invasive pulmonary aspergillosis (IPA) is commonly considered to be defined by invasion of the pulmonary parenchyma by the growing hyphae of *Aspergillus* [2] and this is further refined being angioinvasive IPA if there is evidence of vascular invasion by the hyphae [2]. The most common aetiological agent of IA, *Aspergillus fumigatus,* is a ubiquitous fungus (Figure 1), with airborne conidia leading to almost universal and constant exposure in almost all humans. The corollary of this is that as the risk of IA is mainly a function of deficits in host defences, IA is seen primarily in patients with haematological malignancy and in solid organ and stem cell transplant recipients [3]. Diagnosis of IA is complicated by the fact that unlike many infections blood culture is almost always negative for *A. fumigatus *[4] and sampling of the lower respiratory tract by bronchoalveolar lavage in order to culture the fungus is also insensitive [5]. This lack of diagnostic tools has led to an explosion in the development and evaluation of nonculture diagnostic approaches including imaging, PCR-based detection of *Aspergillus *DNA, and antigen detection—particularly the detection of galactomannan in serum and bronchoalveolar lavage [6]. The difficulties in making a diagnosis of IA lead initially to considerable variation in how cases were defined [7]; however, in more recent years, criteria for the identification of IA have greatly benefited from the European Organisation for the Research and Treatment of Cancer (EORTC) and US Mycoses Study Group (MSG) criteria for defining invasive fungal infections including IA [8, 9]. Though it has been noted that whilst these criteria have been readily taken up, lack of compliance with them even in studies purporting to use them as reference standards continues to hamper comparisons of diagnostic investigations [10]. Nouer et al. [11] have suggested a modified version of these criteria specifically for IA. Together with the development of improved diagnosis of IA, there has been a steady increase in the numbers of licenced antifungals effective against IA [12]. This has resulted in an overall significant improvement in outcomes of patients with IA [13, 14]; however, this has been effected in part through the extensive use of empiric antifungal therapy [15]. The challenge for the current day is to identify diagnostic strategies that reliably detect or predict IA in order to target therapy and improve outcomes or sustain existing outcomes without the need for unnecessary empiric therapy.

### 1.1. Site of Disease

IA is predominantly a disease of the respiratory tract in most cases involving the lung parenchyma [2] but the disease may also involve the pleura and the trachea and bronchi [16]. From these sites, dissemination is reported to occur from pulmonary sites in 10%–25% of cases, particularly to the brain and also including liver, kidney, gut [17], and skin [18]. The sinuses are also an important site for aspergillosis [19] and there has been a recent review drawing attention to skull osteomyelitis following invasive otitis externa [20].

### 1.2. Aetiology of Disease

The most common cause of all forms of IA is *A. fumigatus.* This is one of the most common species in the environment and this fact together with its small spores provides greater penetration to the small airways and ability to grow at 37°C account for its predominance as causal agent [21]. *A. flavus *is the second most common causal agent followed by *A. niger *[22]. However, cases caused by *A. terreus *are seen commonly in some centres [23, 24]. *A. flavus *is frequently seen in nonpulmonary diseases and in developing countries [25, 26]. A new species, *A. tanneri*, that causes invasive disease in patients with chronic granulomatous disease has recently been described [27]. There has been a recent recognition that amongst isolates of some species of *Aspergillus* there are in fact mixtures including cryptic species that can often only be distinguished by DNA sequence analysis. Within isolates thought to be *A. fumigatus, *species such as *A. lentulus*, *A. novofumigatus*, and* A. fumigatiaffinis* and the teleomorphic species* Neosartorya udagawae *have been found upon close examination [28, 29]. Species such as *A. tubingensis *and *A. awamori *have been found amongst clinical isolates previously identified as *A. niger *[30]. These distinctions may seem as taxonomic niceties but these newly recognised species are often associated with distinct patterns of susceptibility to antifungals (Table 1) [30, 31].

### 1.3. Epidemiology of Disease

IA has traditionally been a disease associated with patients with reduced levels of neutrophils, an observation made in the early 1970s [32] together with the finding that neutrophils were capable of the killing of *Aspergillus *hyphae *in vitro* [33]. Thus, IA has mainly been reported as a disease of patients with neutrophil deficits resulting from myeloablative chemotherapy for haematological malignancy and as part of conditioning for stem-cell transplantation [34–37]. While often grouped together for analysis, SCT and acute leukaemia patients may actually present with distinct forms of IA with consequences for optimal diagnostic approaches [38]. The incidence of IA in haematological malignancy varies markedly from 1.7% in a recent study from Italy [39] to nearly 30% in a Dutch study [40] and will be affected by intrinsic factors including recently recognised genetic predisposition to aspergillosis together with the use of antifungal prophylaxis and the extent to which systemic diagnostic screening is performed. Solid organ transplantation, particularly lung and liver transplant, also poses a significant risk for IA [41–44]. Corticosteroid use, particularly during SCT has been recognised as an important risk factor for IA [45]. In recent years, groups of nonneutropenic patients have been shown to at increased risk of IA [46, 47] including patients with chronic obstructive pulmonary disease (COPD) [48], severe liver disease [49], patients in intensive or critical care [50, 51], patients suffering from influenza with H1N1 virus [52], and following surgery [53]. Patients with HIV/AIDS are typically at low risk of IA as immune defect is in CD4 cells which do not appear to play an important role in combating aspergillosis; however, cases of IA in AIDS have been reported [54]. Patients with chronic granulomatous disease (CGD) are at risk of a peculiar form of IA often presenting as fulminant pneumonitis [55]. Genetic factors affecting susceptibility to IA are beginning to be understood and examined. People with a mannose-binding lectin deficiency and mutations in some Toll-like receptors are likely to be at higher risk of IA [56, 57].

### 1.4. Outcomes in IA

The consequences of the development of IA in patients improved markedly in the last 20 years. In 1990, Denning and Stevens surmised that mortality from IA in SCT was greater than 94% [58]. In 2009, results of a prospective antifungal therapy (PATH) registry study indicated that 12-week mortality of patients with HSCT was 35% [14]. A European study of patients with haematological malignancy showed that 12-week mortality was 42% and had declined over the period of the study between 2004 and 2009 [59]. A study of pediatric patients with IA reported a 3-year survival of 55% [60]. However, succumbing to IA remains a factor decreasing the short- and long-term chance of survival. In a study of patients with acute myeloid leukaemia having IA reduced chances of survival at 2-years from 32% to 14% [61]. Analysis of hospital discharge and other medical data in the US has shown that, in general, IA is associated with significantly higher levels of mortality, hospital costs, and length of stay [62–64] compared to similar patients without IA.

### 1.5. Diagnosis of IA

The signs and symptoms of IA are generally nonspecific, and typically involve failure to respond to antibacterial therapy given empirically for fever. Biochemical markers of inflammation such as C-reactive protein or procalcitonin are also nonspecific, though they may have value in monitoring the success of treatment once a diagnosis is established [65, 66]. Approaches to making a specific diagnosis IA can be categorised as involving: imaging, direct microscopy, histopathology, culture, antigen detection, and DNA detection (Table 2). 

Since the work of Caillot et al. in 2001 showed both the specificity and limitations of the high resolution computerised tomography (HRCT) scan of the chest with the signs of the halo and later the air crescent, HRCT has formed an essential investigation of all patients suspected of having IA [67]. Importantly, the halo sign has been recognised as specifically linked to angioinvasive IA [68]. Other signs such as “tree in bud” and segmental consolidation have been recognised as associated with airways disease as compared to angioinvasive aspergillosis [69]. However, studies of 70 patients with IA or pulmonary lymphoma highlighted the difficulties in relying purely on CT imaging to diagnose IA where no single feature provided sufficient sensitivity or specificity [70], and a study of the HRCT signs produced by IA, candidiasis, and cryptococcosis showed a lack of specificity of the halo or air crescent signs for IA [71]. 68Ga-labelled iron siderophores visualised in positron emission tomography (PET) showed promise in rat model of IA as a potential specific imaging technique [72]. This paper will focus on laboratory markers of IA.

One of the problems faced with any disease, where markers have been used to define cases of a disease, is how to make comparisons with newer markers. This has been particularly problematic with IA and has for example restricted the analysis of the galactomannan (GM) test applied to serum where it is often used to define probable IA. It has, for example, only been possible to analyse the value of bronchoalveolar lavage (BAL) GM results in the smaller number of patients who have proven IA where disease is defined by the presence of fungal hyphae and culture in tissue biopsy material [73]. 

## 2. Direct Microscopy

**Figure 2:**
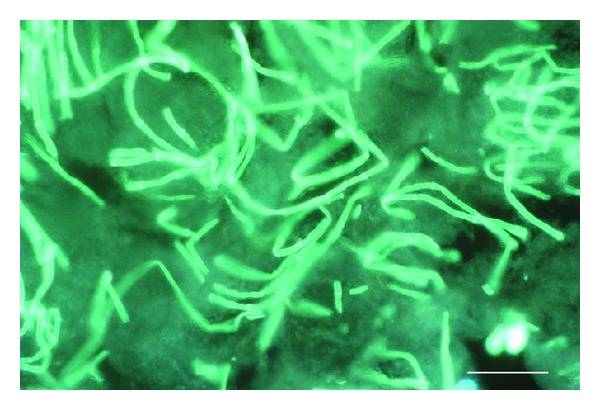
Calcoflour stained tissue from a wound infection that grew *A. flavus.* Bar = 10 um.

One of the simplest approaches to diagnose IA is to examine appropriate specimens microscopically. However, this approach is inherently lacking in specificity as *Aspergillus *sp. rarely sporulate *in vivo *and hyphae seen may represent any number of filamentous fungi. The reported sensitivity of this approach is quite variable from 0% to 90% [74–76] which may reflect differences in detailed methodology such as use of calcoflour (Figure 2) [77].

## 3. Histopathology

**Figure 3:**
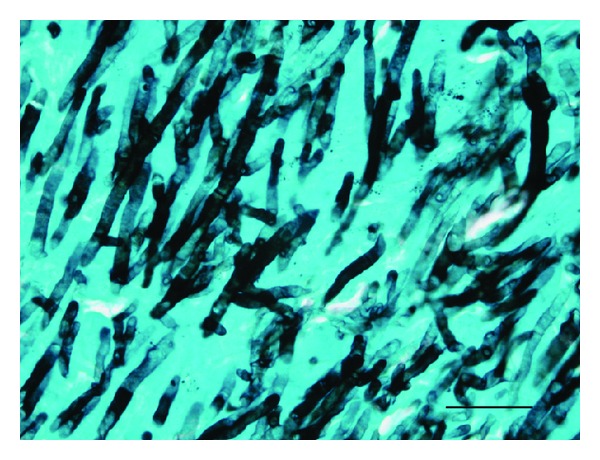
Grocott's silver stained infected lung tissue showing the black hyphae of *Aspergillus *invading lung tissue that is counterstained blue-green. Bar = 10 um.

Demonstrating tissue invasion by a filamentous fungus through histopathological examination of biopsy or autopsy material provides a diagnosis of proven invasive fungal infection (IFI) [78]. Since it is not usually possible to identify a fungus from mycelium seen in tissue sections, positive culture of *Aspergillus *from the same specimen is required to make a proven diagnosis of IA. The value of proven IA is high but in most cases obtaining biopsy samples, in the case of pulmonary IA by transthoracic biopsy is often contraindicated due to low platelet counts or other medical risk. In one case series, only 2/5 patients sampled by transbronchial biopsy were found to have hyphal invasion of the tissue sample, the other 3 were diagnosed as having probable IA based on positive serum and/or BAL GM [79]. Careful examination of tissue sections by standard haematoxylin and eosin staining should reveal the presence of *Aspergillus *hyphae but stains such as period acid Schiff and Grocott's silver may add sensitivity and should be carried out whenever a fungal infection is suspected (Figure 3) [80]. *Aspergillus *hyphae seen in tissue sections tend to be narrow (1–3 um in diameter) and septate and cannot easily be distinguished from a number other fungies that cause infections including *Fusarium *and *Scedosporium*. It is usually easy to distinguish *Aspergillus* hyphae from the wider and pauciseptate hyphae of the mucoraceous moulds such as *Rhizopus *and *Lichtheimia *sp. (histPath book). Some authors have reported methods of identifying fungal cells in fixed tissue sections by immunohistochemical labelling [81]. These techniques are technically demanding and together with the fact that the clinical material to apply these methods is likely to be infrequently sent for investigation means that these methods are unlikely to become popular. PCR identification of hyphae seen in fixed tissue specimens may be able to provide a method of identification if culture of the tissue specimen has not been performed [82].

## 4. Culture

Obtaining a positive culture of *Aspergillus *from a clinical specimen is a traditional way of making or contributing to the making of a diagnosis and prior to the use of GM and PCR was the main specific laboratory investigation for IA [83]. A positive culture will also enable other forms of analysis such as susceptibility testing. In technical terms, most *Aspergillus *sp. grow relatively rapidly (typically within 48 hr) and on most microbiology media including both mycological media such as Sabouraud's agar and blood agars used for general bacteriological culture. Mortensen et al. claim to be able to increase the number of positive cultures by 17% by extending incubation time from 2 to 5 days [84]. While the technique of culturing specimens is inherently simple and low cost, an enhanced method of sensitively, rapidly, and specifically detecting digitonin immobilised microcolonies of *Aspergillus* using the enzymatic cleavage of a fluorescent compound has been described [85]. 

Identification of cultures of most species *Aspergillus* is generally straightforward [De Hoog atlas] by colony and microscopic morphology, though atypical forms, for example, *A. fumigatus*, have been known for many years [86]. More recently, atypical forms of *A. fumigatus* have been re-identified as new species including *A. lentulus, A. novofumigatus*, and *A. fumigatiaffinis* and these require DNA sequence analysis to be reliably identified [28, 29]. 

Using the EORTC criteria, in host and clinical (imaging) factors, positive cultures of *Aspergillus *from respiratory specimens are assumed to represent infecting fungus [87, 88]. In patients where a positive culture is not supported by both host and clinical factors, such cultures are typically interpreted as colonisation [89, 90]. The main problems described for the culture of respiratory and other specimens for the diagnosis of IA are the lack of sensitivity and the difficulty in distinguishing between infection and colonisation. The percentage of neutropenic patients diagnosed with IA by GM positive BAL who are positive by culture ranges from 10% to 58% [91–100]. In a study of the performance of a PCR and the GM assay on BAL samples mostly from haematology patients, in 17 cases of proven or probable disease where the mycological component of the diagnosis was by serum GM, only 7 were culture positive [101]. Failure to culture *Aspergillus *from respiratory specimens may lead to the incorrect assignment of *Candida *sp. as the aetiological agent from positive cultures and this may have led to the suggestion that HRCT signs previously thought of as pathognomic for IA are being also attributed, probably erroneously to invasive candidosis [102]. In a rabbit model of IA, culture of BAL varied in sensitivity in untreated animals from 50% to 100% depending on the infecting *Aspergillus *sp. [103]. *A. fumigatus *infected rabbits treated with amphotericin B became completely culture negative whilst signal was still obtained from GM or PCR testing of BAL, indicating the advantage of culture in terms of detecting viable fungus. 

## 5. Galactomannan Detection

Galactomannan (GM) is a carbohydrate molecule composed of a backbone of mannose residues with side chains of *β* 1–5 linked galactofuranosyl residues. Detection of this molecule was described by Stynen in 1992 using a monoclonal antibody (EB-A2) [104] which specifically binds to four galactofuranosyl residues leading to the suggestion that GM, as detected by EB-A2 in the diagnostic ELISA which was later developed, is better referred to as galactofuranose [105], though this terminology has not been widely adopted. Pure GM is readily released from *Aspergillus *sp. growing *in vitro *as a molecule of about 20 kDa in size, but it is also present in larger molecules linked to proteins as glycoproteins [106, 107] and a lipopeptide galactomannan [108]. GM is part of the cell wall along with chitin, *β* 1–3, and 1–4 glucan [109], and while the GM is likely to be incorporated during periods of logarithmic growth this is also the stage of growth *in vivo *at which GM release peaks [110, 111] presumably because of leakiness in the hyphal tip during active growth. The extent to which active logarithmic growth occurs during infection *in vivo *is unclear but may be restricted by lack of nutrients particularly in necrotic tissue. The release of GM glycoproteins is thought only to occur during cell wall lysis which may occur *in vivo *where nutrients are limited and growth also is restricted by the host response and in necrotic oxygen deprived tissue [112]. The level of EB-A2 positive material during growth *in vivo *also appears to relate to the secretion of beta-galactofuranosidase which degrades the galactofuranosyl epitopes of this antibody [113, 114]. From an elegant *in vitro *model of the invasion of lung tissue where the release of various components in alveolar and endothelial compartments can be detected, it has been suggested that GM is not released into the circulation during infection until the fungus invades the endothelial compartment [115]. This suggests that circulating GM cannot be detected until angioinvasion by the fungus occurs. Release of GM into the circulation during angioinvasion is supported by the observation that serum GM is almost always readily detected in neutropenic haematological and SCT patients where angioinvasion is seen in characteristic halo signs in HRCT [116]. In contrast, in patients with chronic granulomatous disease, IA is often characterised by lung abscess development which may limit angioinvasion and patients are frequently serum GM negative [117]. Once in the circulation, levels of detectable GM are likely to be subject to clearance through the kidneys and by active uptake by macrophages [118]. Antibodies to GM can also develop which have been suggested as a cause of false negative serum GM results in patients with IA [119].

### 5.1. The GM Assay

The original assay for GM was a latex agglutination LA test with a limit of detection of 15 ng/mL, which was soon replaced by a sandwich ELISA able to detect less than 1 ng/mL [120], and most of the literature where the assay is used relates to the ELISA assay. The original specimens used in the GM assay were serum and urine. GM which can be detected in the urine of patients with IA [121, 122]. Animal models suggest that excretion of a significant proportion of GM occurs in the urine [118]. Early reports suggested that in human IA urine was a more sensitive specimen than serum [123], which was contradicted by the findings of others [124]. Furthermore, there is concern that GM is more likely to be detected in the urine of patients who are further down the course of invasive disease and where interventions are less effective [125]. The variety of methods used in these early studies, together with differences in pretreatments, mean that the value of urine as a specimen for GM detection remains unclear [126] and urine testing for GM is rarely used in recent studies. In contrast, the interest in BAL as a specimen for GM analysis has increased markedly since original observations of the presence of GM in BAL fluid [127] and BAL along with serum are currently the only specimens approved by the manufacturers for analysis. BAL is an attractive clinical material for analysis of pulmonary IA, as it has been shown that GM is released during hyphal growth rather than from conidia [128, 129] thus potentially enabling the distinction between colonisation and active infection. The difference between the yield of bronchial lavage (BL) as compared to BAL has been investigated and some have suggested that BL is more sensitive than BAL for the GM test, though conversely BAL was more sensitive for culture [130] though this has not been confirmed in subsequent studies [131]. A series of studies in patients with haematological malignancies and undergoing stem cell transplantation [91, 132–138], lung, and other solid organ transplant recipients [89, 99, 139] and patients in critical care [140, 141] have confirmed the value of BAL as a specimen for GM. GM has also been detected in lung abcess fluid [142]. More recently, it has been reported that detection of GM in sputum in cases of IA in haematological malignancy may prove useful [143]. Using a cutoff of 1.2 100% sensitivity and specificity for sputum was obtained compared to values in BAL of 62% and 83%, respectively, and this may prove a valuable less invasive sample to test. Cerebrospinal fluid (CSF) is another specimen type used for the detection of GM during attempts to diagnose central nervous system IA. The brain is the most common site for dissemination from the lungs. CSF may not be thought to be the most appropriate specimen for the diagnosis of central nervous system IA which tends to present as cerebral abscess and culture of CSF is rarely positive in cerebral IA [144]. However, GM has been detected in several cases of CNS IA [145]. In a study of 5 bone marrow transplantation patients with cerebral IA, the mean GM level in CSF was positive and higher than that in patients without IA [146].

In the commercial kit, there is a pretreatment step with an acid EDTA solution and heating in order to precipitate proteins and break up immune complexes. This may reduce the activity of the acid labile furanosyl side chains of GM and any galactofuranosyl moieties of glycoproteins may also be lost in this step [147]. It has been suggested that a microfiltration concentration step can be used to increase analytical and clinical sensitivity [148]. Some authors have described problems with the reproducibility of the assay [149]. Recently a monoclonal antibody that detects GM-like antigens was derived and used to develop a lateral flow assay to revisit the potential for diagnosing IA by detecting GM in urine [150]. 

### 5.2. Galactomannan Is Not Unique to *Aspergillus* sp

GM is found in varying amounts in other fungies including *Penicillium, Fusarium, Alternaria*, and *Histoplasma* [151–153]. This presents a variety of problems. The ability to detect *Penicillium marneffei, *the only pathogenic *Penicillium *species, through GM detection is unlikely to result in clinical dilemmas since *P. marneffei *infections are seen in very distinct patient types compared to IA, that is, HIV with travel history to Southeast Asia [153]. On the other hand, cases of histoplasmosis seen in solid organ transplant recipients where serum and BAL specimens were positive for GM can create difficulties in patient management and these results are often referred to as false positives [154]. Similarly, patients with systemic *Fusarium* infection, seen classically in haematological malignancy, have also been found to be positive for the GM test though some authors take a more positive view in terms of the use of this assay for the diagnosis of *Fusarium *infection [155, 156]. Perhaps, most controversially, some authors have claimed that antigens from *Cryptococcus neoformans *cross-react in the commercial GM test and positive reactions seen in patients with cryptococcosis [157]. Positive results have also been reported for infections cause by other yeasts such as *Geotrichum capitatum *[158]. Swanink et al. who surveyed a range of fungi for cross-reacting antigens suggested that *Candida *sp. did react in the GM assay at a low level [159], and when sufficient numbers of yeast cells are processed, positive signals can result (R. Barton, unpublished data), a finding that have unlikely an impact on serum specimens but might result in false positives in BAL samples.

A more important problem resulting from the presence of cross-reacting antigens in *Penicillium *is the presence of GM in preparations of some antibiotic agents. False positives resulting from the use of piperacillin tazobactam have been reported since 2003 in patient sera and confirmed on testing antibiotic preparations [160, 161]. Recent investigations into the presence of GM in tazobactam from different manufacturers suggest that while there are still manufacturers whose antibiotic continues to be associated with false positive GM results [162], the problem has largely abated [163]. Other sources of false positive GM results include nutritional supplements [164, 165], and there have been theories of other foodstuffs including milk [166] and pasta [167] where cross-reacting antigens are thought to translocate from the gut to the blood and cause false positives, particularly in cases of gut mucosal disturbance during chemotherapy and in children. Most recently, galactomannan has been found in preparations of electrolyte solutions such as PlasmaLyte. Gluconate in some of these preparations is derived from *A. niger*, and GM from *A. niger* is present to the extent that it may cause false positive reactions [168–170].

One of the most carefully investigated cross-reacting antigens are those found associated with gut bacteria *Bifidobacterium* sp. Mennink-Kersten et al. hypothesised that lipoteichoic acids associated with the cell wall of *Bifidobacterium *sp. bind to the EB-2A monoclonal used in the GM ELISA [171] and that this was linked to the high rate of false positives seen in children and neonates. This was followed up by the demonstration of the cross-reactivity of almost all *Bifidobacteria *sp. tested in the GM assay and the presence of these bacteria extensively in neonatal faeces [172]. This theory was prompted by findings of the relatively poor specificity of the GM assay in neonates and children. Siemann et al. reported that in 5/6 premature neonatal patients aged between 19 and 136 days, sera were strongly positive for galactomannan, and in 3 cases sera tested positive repeatedly [173]. However, this is the only evidence for the problem of specificity in neonates, this is possibly because neonates are not a major at risk group for IA and are rarely tested. In general, specificity in slightly older paediatric patients is >90% [174–177]. 

### 5.3. Clinical Validity of GM Testing

A systematic review of serum GM testing for the diagnosis of IA was published in 2006 by Pfeiffer et al. [178]. Twenty-seven studies published between 1999 and 2005 were included and the main analysis was in patients with proven disease, defined by histological evidence of tissue invasion, reducing incorporation bias as the GM test itself can be used to define probable IA according to EORTC/MSG criteria [8, 179]. The overall sensitivity of the test for proven IA was determined to be 71% and specificity 89%. The limitation of this analysis was that at the time when most of the studies were published, a cutoff of 1.5 or 1.0 was being used and only 5 studies provided using this cutoff of 0.5, which is now recognised and used as a standard. In the studies, by using the 0.5 cutoff, ironically a sensitivity of 27% and overall the accuracy of the test improved with increasing the cutoff. Significant heterogeneity was observed amongst studies, particularly in sensitivity as had earlier been noted by Leeflang et al. [180] and attention was drawn to the lack of sensitivity in studies of patients with solid organ transplantation (22%). Importantly, there was little difference in the performance of the test between adults and paediatric patients. 

In 2008, Leeflang et al. published a Cochrane review and meta-analysis of the GM assay on serum specimens including 30 studies between 1998 and 2007 [180] and included in analysis either proven or probable cases where the mycological criterion was defined by an alternative method to serum GM, for example, a positive BAL culture. Again, a minority of these studies, seven, used a cutoff of 0.5 and in this subset a sensitivity of 79% and specificity of 82% were obtained. It has been noted by other authors (Table 2) [181] that the incidence or pretest probability will affect the value of the GM (and any other test), particularly the positive predictive value. Leeflang et al. noted that at a prevalence of 8% of IA as seen in their meta-analysis, applying the test with a cutoff of 0.5 would lead to missing a diagnosis of IA 2% of patients and treating 17% of patients unnecessarily in a population of at-risk patients [180]. Again significant heterogeneity was seen though there were small numbers of patients with IA in some studies. A recent meta-analysis of 34 studies of the use of serum GM to diagnose IA between 1991 to 2008 suggested that the pooled sensitivity was as low as 66% and specificity was 90% [182]. Again significant heterogeneity was observed. At the incidence of IA observed of 10%, rates of under diagnosis and misdiagnosis were higher at 34% and 10%, respectively. Studies of the efficacy of serum GM to diagnose IA published more recently between 2009 and 2012 suggest that authors findings on the proportion of patients with IA that are positive for GM continues to vary ranging from 13% to 87.5%. The specificity of the assay on serum tends to be higher ranging from 21% to 100% and in most cases greater than 80%.

The most carefully analysed variable is the effect of mould-active antifungal therapy, typically for prophylaxis, on the sensitivity of the serum GM assay. Marr et al. analysed data for 317 immunocompromised patients finding overall sensitivity of the serum GM assay to be 70% for proven and probable IA, where the GM test itself was not used to define disease, and at a GM cutoff of 0.5 [186]. However, the sensitivity of the assay in patients receiving antifungal therapy (mostly itraconazole) on the day of diagnosis dropped to 52% compared to 89% in patients not receiving antifungal therapy. In other words, in this study, in patients on mould active antifungal therapy with breakthrough IA, serum GM was only effective in diagnosis in about half of the cases [187]. This result could be logical where antifungal treatment inhibits growth of *Aspergillus *or the cells renders nonviable reducing the load of fungal cells able to shed the GM antigen. Certainly animal models of IA have shown that the burden of *Aspergillus *as measured by lung chitin and galactomannan correlates with the level of circulating GM [188]. Similarly *in vitro *models of treatment appear to correlate reduction in fungal load with galactomannan [115]. The effect of the timing of the exposure of the antifungal exposure relative to the establishment of the inoculum is clearly critical. In the relatively unsophisticated model of Winn et al. where antifungal was added to germinating *A. fumigatus *conidia and GM release monitored, growth and GM levels were suppressed [189]. However, Hope et al. in their two compartment model, showed that delaying the amphotericin treatment of the same dose of the *Aspergillus *inoculum by six hours resulted in a failure to suppress GM release into the endothelial compartment which models the release of GM in the blood stream following pulmonary disease [115]. It has also been suggested that actively growing *Aspergillus *hyphae exposed to antifungals might increase the release of GM. Petraitiene et al. [190] described a rabbit model of IA treated with caspofungin where antifungal therapy resulted in an increase in GM despite improvements in terms of animal survival [190]. However, this increase in GM levels was again associated with increased fungal load, a function of the so-called paradoxical effect of echinocandin antifungals. However, in breakthrough cases of IA, perhaps due to resistance in *Aspergillus* or suboptimal drug levels during prophylaxis, where in order for a disease to become apparent, fungal burden must presumably increase, it remains to be seen how *Aspergillus* exposed to antifungal agents might result in infections where less GM is shed into the blood.

Another possible cause of the variability in sensitivity of the serum GM assay is the level and nature of immune suppression of the patient. Classically, the assay has been applied to patients with haematological malignancy or having undergone bone marrow or stem cell transplantation, both groups with low neutrophil counts. Cordonnier et al. have recently shown that amongst haematology patients with neutrophils levels <0.1 × 10^9^/L are more likely to have higher serum GM levels than patients with higher neutrophil levels [191]. The serum GM assay has been shown to be modestly sensitive in COPD patients with IA [192, 193] and poorly sensitive in solid organ transplantation [194, 195] though more recent studies have achieved greater sensitivities in this patient group [196]. The lower incidence of neutropenia in these two patient groups may well account in part for the reduced sensitivity of the serum GM assay. Serum galactomannan was shown to have lower sensitivity in patients who had antibodies to *Aspergillus fumigatus *[119]. Finally, there are a number of laboratory parameters that will affect the probability that a patient is scored positive for a serum GM test including the frequency of testing, whether one or two positives are used to define a case of IA and the cutoff used. Some of the studies with the highest sensitivity have involved at least twice weekly sampling [197, 198]. There is a clear relationship between the cutoff used to define a positive score in the GM assay and the sensitivity. The original Bio-Rad Platelia assay recommended a cutoff index for positivity of 1.5, with indices of 1–1.5 considered an equivocal zone [199]. Currently, a cutoff of ≥0.5 is positive and <0.5 is negative. Meta-analysis showed that the sensitivities at 1.5, 1.0 and 0.5 cutoff are 62%, 71% and 79%, respectively [180]. A later meta-analysis [200] showed, unsurprisingly, that requiring two positive sera to define IA reduced sensitivity and increased specificity.

### 5.4. Clinical Validity of BAL GM

Guo et al. [184] have analysed the present considerable literature on the use of BAL as a specimen for the GM assay. Thirteen studies between 2003 and 2009 were considered and an overall sensitivity of 90% and specificity of 94% were calculated. A more recent meta-analysis of 30 studies gave figures that were slightly lower (87 and 89%, resp.) (Table 3) [201]. This sensitivity is notably higher than that seen in many studies of serum GM and the diagnostic odds ratio is notably higher than all blood-based tests. Analysis of 33 cases of IA where a blood sample and BAL specimen were taken within a week and where one or both were positive showed that all 33 BAL specimens had positive GM indices >0.5 compared to 9/33 (27%) of the sera [202]. Receiver operator characteristic (ROC) curve analysis of GM data from BAL and serum in a series of patients with proven or probable IA showed higher areas under the curve (AUC) for BAL testing compared to serum [141]. One of the observations that most studies note is that the background level of GM or EB-A2 positive material in BAL is greater than in serum [203]. This has raised the question of what is the appropriate cutoff for the assay applied to BAL material. The manufacturers of the kit, Bio-Rad, recommend the same cutoff as in serum, that is, >0.5 indicating a positive score [199]. However, values of 0.5 to 2 have been suggested [184]. Analysing the results of BAL GM testing in patients with haematologic disease showed that increasing the cutoff between 0.5 and 4 increased the positive likelihood ratio from 5 to 25 [204]. Using ROC curve analysis on 85 BAL GM data from paediatric patients at risk of IA, Desai et al. suggested that an optimum cutoff would be 0.87 giving a sensitivity of 78% and specificity of 100% [205], and a similar analysis leads Desai et al. [205] to propose a cutoff of 0.8. Park et al. 2010 [206] have even suggested using a cutoff of 0.2, and though applying this to a cohort of 359 at-risk patients gave a sensitivity and specificity of 86%, and 74%, respectively, a cutoff this low in a specimen that tends to have high background levels of GM is likely to lead to large numbers of false positives in most settings. Although the specificity of BAL GM testing is typically high, Acosta reported an intensive care patient with high levels of BAL GM without corroborating evidence of IA, direct microscopy and culture were negative, and who recovered without antifungal therapy [141]. *Penicillium* sp. colonisation may result in false positives [207, 208].

## 6. Detection of 1,3 **β**-D-Glucan (BDG)

The cell walls of *Aspergillus* contain relatively large amounts of glucan of which 1,3 *β*-D-glucan forms a large part [209]. *In vitro* analysis of growing *A. fumigatus *showed that, like GM, BDG is released during logarithmic growth, though slightly later [210]. Unlike GM, BDG is widely distributed in the fungal kingdom and is found in the cell walls of many pathogenic fungi including *Candida*, *Fusarium*, and *Pneumocystis* though it is present at a lower level in *Cryptococcus* and virtually undetectable in the mucoraceous moulds [211]. Similar to GM, BDG is excreted into the culture fluid of *A. fumigatus *[212, 213].

### 6.1. The 1,3 *β*-D-Glucan (BDG) Assay

BDG can be detected through a pathway in the *Limulus *amoebocyte lysate (LAL) coagulation cascade that has traditionally be used for the detection of bacterial endotoxin. Whilst endotoxin interacts with LAL via factors B and C, factor G in the LAL interacts with BDG activating a proclotting enzyme which can then cleave a chromogenic substrate to generate a product detectable by spectrophotometry down to 10 pg/mL [214, 215]. Animal models of IA [216] and patients with IA [214, 217] were shown early on to have often high levels of BDG in serum validating BDG detection and as a potential diagnostic marker. Using pre-EORTC criteria of 30 definite or suspected pulmonary IA cases, 63% were positive by BDG using a cutoff of 20 pg/mL [218], and 73% of 185 control patients were negative. Patients with other fungal infections have been found to be positive for the BDG assay including those infected by *Candida, Fusarium *sp., *Acremonium *sp., *Pneumocystis*, and *Histoplasma capsulatum *[217, 219–221]. Thus, the BDG assay has emerged as a generic marker for invasive fungal disease rather than a specific marker for IA. 

Several authors have questioned the specificity of the BDG assay further. Evidence of false positive results due to cellulose haemodialysis membranes [222], intravenous immunoglobulins [223], and antibiotics such as amoxicillin-clavulanic acid [224] has been described. In 2003, Digby et al. found that increased levels of BDG were seen in infected ICU patients both with bacterial and fungal infections compared to noninfected ICU patients [225]. Though the patients in this study were not defined according to EORTC criteria, and insufficient detail on the bacterial and fungal isolates in question was given to properly assess this report, it did suggest that the BDG assay was not specific for fungal infections. Pickering et al. [220] found that 10 of 14 patients with gram positive bacteremia without evidence of invasive fungal disease were BDG positive. However one patient had undergone haemodialysis and other causes of false positives such as the use of cotton gauze could not be ruled out. Based on reactivity of culture supernatants, it has been suggested that patients with *Pseudomonas aeruginosa *infections may be at risk of producing false positives [226]. In general, these observations of bacteremia resulting in false positives of the BDG test have not been corroborated [217, 227, 228]. Specifically, Racil et al. have examined sera from 26 high risk haematology patients with bacteremia and only 2 were positive for BDG and both of them had invasive fungal infections [229], and Metan et al. [230] found that only 1 of 14 bacteremic patients positive for BDG had no evidence of an invasive fungal infection suggesting that bacteremia is in fact a rare cause of false positives.

### 6.2. BDG Assays

There are four different commercial assays for the detection of BDG in clinical specimens. The Associates of Cape Cod Fungitell kit uses amoebocyte lysates from *Limulus polyphemus* while the Seikagaku Fungitec-G test uses reagents from *Tachypleus tridentarius* as does the Wako *β*-Glucan test [211]. Recommended cutoffs for reporting positive results vary between assays. Manufacturers of the Fungitell assay requires >80 pg/mL to be detected for a positive with 60–79 pg/mL termed intermediate based the work of Ostrosky-Zeichner et al. [231]. Cutoffs for the *T. tridentatus *based assays are lower at 20 pg/mL [217] and even lower cutoffs of 11 pg/mL or two sera at 7 pg/mL have been proposed [228]. A fourth assay, GKT-25-M has been reported in the Chinese literature [185].

### 6.3. Validity of the BDG Assay

A meta-analysis of serum BDG diagnostic accuracy identified 31 studies of invasive fungal infection between 1995 and 2011 for analysis including 17 studies where IA was specifically targeted or identified as a subgroup for analysis [185]. This analysis estimated that the sensitivity for the detection of IFIs was 80% and the specificity was 82%, whilst for IA the figures were 77% and 83%, respectively (Table 2). Significant heterogeneity was found in this analysis. Odabasi et al. [227] examined BDG levels using the Glucatell assay in haematology patients. They proposed a cutoff of 60 pg/mL based on 30 candidemia patients compared to 30 controls, and four patients with *Aspergillus* pneumonia or fungemia all had BDG levels greater than 60 pg/mL. Ostrosky-Zeichner et al. [231] performed a multicentre study of 163 patients from diverse specialities and invasive fungal disease of whom 22 were diagnosed with proven or probable IA. Based on this dataset, they proposed increasing the cutoff to 80 pg/mL which provided a sensitivity of 64% and specificity of 92.4%. This has been confirmed as a reasonable level by others [230]. Senn et al. [228] looked at neutropenic leukaemia patients and found 15/32 IA cases amongst those with proven or probable fungal disease. Though they did not analyse the IA cases separately, using the Wako *β*-Glucan test and cutoff of 7 pg/mL in two consecutive sera a sensitivity of 63% and specificity of 96% were obtained for invasive fungal disease overall. Using two consecutive sera was backed up by ROC analysis with the area under the ROC curve for 2 sera being 0.87. Comparisons of the BDG and GM test on sera suggest the tests are in most cases comparable for the diagnosis of IA [141, 232, 233], though some studies suggest that BDG may be more likely detected earlier than GM during the development of infection [234]. The BDG assay has generally been applied to haematology patients at risk of IA due to neutrophil deficit, but others have shown the value of this test in intensive care patients [141] and patients with COPD [235]. In an animal model of IA, the BDG assay was applied to BAL as well as serum and was shown in a similar way to GM to both become positive earlier in BAL during the infection course and have a higher sensitivity overall compared to serum [236].

## 7. Detection of *Aspergillus* DNA

Soon after the invention of the polymerase chain reaction (PCR) in 1988, reports were being published of how DNA extracts from BAL specimens could be subject to PCR for the detection of *Aspergillus *DNA and thereby effect a diagnosis in animal models of IA and in patients at risk of IA [237, 238]. Concerns about differentiating between colonisation and infection limited the use of this approach. However, in 1997 Einsele et al. extracted DNA from the blood of neutropenic haematology patients and amplified up part of the large subunit *rRNA* gene using pan-fungal primers detecting *Aspergillus *DNA by dot blotting in all of the 13 patients with IA [239]. Since then, there has been extensive interest in this technique. However, due to the fact that, until recently, there were no commercial PCR assays, the main problem has been the publication of multiple assays with differences in DNA extraction, PCR, and product detection with little or no standardisation that prevented easy comparison of study results [240]. A striking visualisation of this diversity can be seen in 29 different protocols in Table 2 of a recent evaluation of PCR protocols [241]. This lack of standardisation leads to the failure to include DNA detection being incorporated into the EORTC-MSG criteria for the definition of invasive fungal infection [242].

### 7.1. *Aspergillus* DNA

In *Aspergillus *sp., DNA, as in all other fungi, is present in both the nucleus and mitochondria and both have been used as targets for diagnostic assays [243]. The genome of *A. fumigatus* was sequenced in 2005 [244] and has a total size of 29.4 Mbases containing nearly 10,000 genes. Some authors have suggested that unlike GM and BDG, during growth of *in vitro Aspergillus*, DNA does not appear in the culture supernatant until late in the growth phase when the hyphal cells start to autolyse [245] and this also held true for the more sophisticated human cell-based *in vitro *model [115] of Hope et al. However, Morton et al. (2010) [111] using a more sensitive PCR assay found that DNA release did occur during exponential growth *in vitro*.

### 7.2. PCR Assays

The main advantage of molecular detection is that unlike almost all other detection methods other than culture, there is an element of amplification of the *Aspergillus *signal and therefore the method has potentially very high sensitivity. Furthermore, PCR methods for the detection of fungal DNA can also be tailored to detect all or most fungi, or members of the genus *Aspergillus* or a particular *Aspergillus *sp. through primer and probes design. With many viral infections being diagnosed through PCR-based methods, the technology and expertise is present in most clinical laboratories, and consumables costs for PCR tests, once high, have continued to become more competitive.

Although other biomarkers such as GM and BDG could theoretically be applied to any specimen type, in practice it is only PCR-based methods for DNA detection that have been applied to a wide range of clinical specimens. Respiratory specimens such as BAL and sputum represent obvious material as most cases of IA start as, or compromise wholly of, pulmonary disease. It has been shown that in BALs with high cellularity, positive PCR results are more common, perhaps reflecting the presence of phagocytosed conidia and hyphae in macrophages and neutrophils [94]. Due to the difficulty in obtaining multiple routine specimens, detection of DNA in blood samples has been the focus for much research effort. Furthermore, the presence of *Aspergillus *DNA in the bloodstream compared to that in respiratory specimens is considered to be less equivocal in terms of clinical significance [246]. Positive results from PCR testing of blood specimens has led to extensive discussion on the nature of the original DNA signal in the specimen. Viable fungus is, unlikely as *Aspergillus *fungemia, extremely rare [4]. Nonviable *Aspergillus *cells may be present inside phagocytic cells such as monocytes though many of the patients in question are pancytopenic. It has been shown that platelets bind *Aspergillus *hyphae and can reduce viability [247], and platelet-bound *Aspergillus* hyphal fragments may contribute DNA to positive PCR test results. Whether free *Aspergillus *DNA might circulate in blood is not clear though there have been many successful demonstrations of *Aspergillus* DNA in serum specimens from patients with IA where centrifugation of the blood suggests that soluble DNA is being detected [248, 249]. *In vitro Aspergillus *DNA is stable in serum [111], and in animal models it has been determined that *Candida *DNA can be detected at least 2 hours after inoculation [250]. The uncertainty of which blood fraction to use has led to a variety of fractions being recommended for use in *Aspergillus* PCR testing including whole blood [241], serum [251], and even blood clots [252]. There is evidence that whole blood is more likely to test positive for *Aspergillus* DNA than plasma [246, 253]. Most recently a method utilising DNA extracted from both plasma and whole blood provided a very high sensitivity [254]. Other specimens that have been used include CSF [255], tissue, and urine [256, 257].

### 7.3. DNA Extraction Methods

In order to have effective DNA detection, the *Aspergillus *DNA within a clinical specimen needs to be purified as much as possible from inhibitory substances and at as high a concentration as possible for amplification. It may also be useful to purify the *Aspergillus *DNA away from human DNA, as although specific primers and probes will be used for detection, in some PCR assays human DNA in large concentrations can interfere with optimal detection [258]. Broadly, one of the main variations in the method is the initial step where any intact fungal cells are lysed by either enzymic or mechanical means. It has been observed that extraction of DNA from respiratory samples is more straight forward and it has been suggested that a mechanical method such as bead beating is superior for the extraction of *Aspergillus *DNA from BAL material [259], though good results have also been obtained by others using enzyme-based methods [260] and sensitivities of 80.6% and 88.9% have been calculated from reviews of several studies using this approach [246]. A caveat with enzyme-based methods is that some preparations were identified as a source of contaminating fungal DNA in early studies [261]. Extraction from whole blood is more complex, and many methods propose lysis of red and white blood cells prior to fungal cell lysis and further cleaning up and concentration steps [241, 262]. Attempts to use *Aspergillus *DNA enrichment techniques to improve sensitivity have proven unsuccessful [263]. Extraction methods may be manual and quite time-consuming or make use of automated extraction machines though usually some manual preextraction steps are required [264]. Recently, the European *Aspergillus* PCR Initiative (EAPCRI) have, after years of meticulous multicentre evaluation, proposed standards for the extraction of *Aspergillus *DNA from whole blood [241, 265]. A panel of *A. fumigatus *spiked blood samples was circulated to 22 centres and by comparing performance with compliance to the protocol standards requested, the EAPCRI laboratory working party was able to determine that factors including use of a large volume of blood (at least 3 mL), bead beating during extraction, and inclusion of an internal PCR control are essential for sensitive and specific assay. This exercise also confirmed the earlier suspicions that, with certain caveats, effective detection of *Aspergillus *DNA is a function primarily of extraction method and not subsequent amplification. A similar set of proposals for the standardisation of the extraction of *Aspergillus *DNA from serum has recently been published by the same group [251]. One caveat to these recommendations is that these assays have been developed and validated using blood spiked with *Aspergillus *conidia though this is not a valid model since although the exact form of the fungus present in blood is not known, it is highly unlikely to be conidial as *Aspergillus *rarely sporulates *in vivo.*

### 7.4. Amplification

All PCRs require a pair of oligonucleotide primers that will bind and prime synthesis of a short stretch of *Aspergillus *DNA. Of the more widely used nuclear DNA, one of the most popular targets are the genes and spacers of the ribosomal RNA because as in all fungi, in *A. fumigatus*, and presumably other *Aspergillus *sp. there are multiple copies though the copy number can vary significantly between strains between 38 and 91 copies [266]. Thus PCRs are to amplify regions of the small subunit (or 18S) rDNA [103, 267–270], large subunit (confusing referred to as 25S, 26S, or 28S) rDNA [248, 271], and intervening transcribed spacers (ITS) 1 and 2 [272, 273]. A multicentre study of PCR assays applied to *A. fumigatus *DNA extracted from conidia added to whole blood and using 18S and 28S targets suggested that detection of the 28S target was more sensitive and specific though in part this effect was platform specific seen with one particular PCR machine [258]. This was thought to relate in part to the fact that the 18S primers amplified a more conserved sequence and human DNA was coamplified affecting optimal detection of the fungal DNA. Single copy genes such as proteinases [238] and ribotoxin genes [274] have also been used for targets. Mitochondrial DNA targets have been compared with rDNA targets [243] and were shown to have slightly lower analytical sensitivity but slightly superior clinical sensitivity. Despite the wealth of resources of published primers, newly designed primer assays continue to be published [275]. In recent years several groups have described assays combining diagnosis with detection of azole resistance in *Aspergillus fumigatus* by targeting the CYP51A gene [276–278].

Whilst the polymerase chain reaction in various forms has been the mainstay of molecular detection technology, nucleic acid sequence-based amplification (NASBA) methods to detect *Aspergillus *RNA have been described [279]. NASBA is considered by some to be more robust than PCR with the advantages of requiring less specimen, using isothermal amplification and is less likely to have contamination problems from fungal DNA in enzymes used for extraction [280]. Real time NASBA methods have been developed [281, 282], and in an animal model compared to PCR [283] NASBA appears to be sensitive. When applied to clinical specimens from patients with IA, detection of total nucleic acids by NASBA has also shown good sensitivity [284]. Morton et al. have investigated reverse transcriptase methods to detect rRNA in blood in a murine model of IA and found this approach to have potential where effective systems for stabilisation and isolation of RNA are used [285].

### 7.5. PCR Product Detection

Real-time (RT) PCR products can be detected either by a generic stain such as SYBR green or more commonly using a specific probe, first described by Loeffler et al. in 2000 [286], and this has become a standard, involving the use of hydrolysis [287, 288] and hybridisation probes [249]. RT PCR has advantages of speed, reduced contamination as no post-PCR manipulation is required, increased sensitivity and specificity, the potential to reduce costs through low volume and low reagent costs, and quantification of signal. However, Johnson et al. [257] have argued that there has a been a lack of detail in published studies on RT PCR methods for the detection of *Aspergillus *DNA, needed to understand the wide range of analytical and clinical sensitivities reported. They advocate the use minimum information for the publication of real-time quantitative PCR experiments (MIQE) and report a RT PCR assay fulfilling these guidelines and demonstrating good performance *in vitro* and on clinical specimens from patients with proven or suspected IA [257]. In an effort to improve both sensitivity and specificity, several authors have described nested-PCR assays where an initial product is subject to a second round reaction with new primers [289, 290]. However, most authorities regard this approach as flawed as it requires post-PCR manipulations and increases the risk of contamination and false positives, though studies using nested-PCR methods continue to be published [255, 291]. Many PCR assays only target *A. fumigatus*, the most common species. In order to ensure that all species of *Aspergillus *are detected, one approach is to amplify a region of DNA conserved in *Aspergillus *spp. and to use probes specific for each species [103, 292]; alternatively, products can often be identified using a single probe and melting curve analysis [257, 270]. These approaches are likely to be useful in centres where a significant proportion of cases are caused by non-*fumigatus* spp. ELISA systems for the detection of PCR products have also been developed for *Aspergillus *DNA detection, and though effective, they have been found to be less easy to work with than RT-PCR methods [293]. It may seem remarkable that as recently as 2009 block-based PCR assays with detection by running out products by gel electrophoresis and staining with ethidium bromide have been published [269], though White and Barnes have reported that sensitivities of conventional and real-time PCRs are equivalent whilst the specificity of conventional PCR tends to be lower [246].

In recent years, commercial PCR kits have been launched providing standardisation and a high level of quality control in the development and manufacture of the kits. The Roche Diagnostics Septifast is designed to detect a range of microbial pathogens from blood including *A. fumigatus. *In studies of haematology patients, *A. fumigatus *has been detected in blood samples though the diagnostic comparator in these studies was blood culture which was unsurprisingly negative [294, 295]. In a study of three patients with probable IA as defined by EORTC-MSG criteria, all were positive by Septifast for *Aspergillus *DNA. The test was also positive for one patient with no evidence of IA [296]. Case reports of this assay being of use in the diagnosis of IA in liver transplant recipients [297] and a case of *Aspergillus *endocarditis have been described [298]. Myconostica have recently launched the Mycassay *Aspergillus* PCR kit which has been compared with a well-established in-house assay and compared favourably in terms of sensitivity in serum [299], BAL specimens [300], and also lung tissue [301].

As in the case of other serological assays, improving the specificity of PCR tests for *Aspergillus *DNA by requiring two consecutive positive results has been considered [246]. This approach has been shown convincingly to enhance specificity without compromising sensitivity in studies where patients with febrile neutropenia were serially screened [275, 302].

### 7.6. Validity of PCR

A meta-analysis of PCR methods applied to whole blood, serum and plasma to detect IA was published in 2009 [183], examining 16 studies published between 2000 and 2008. Analysis using a single positive PCR gave an overall sensitivity of 88% and specificity of 75% whilst requiring two positive samples reversed the figures with 75% sensitivity and 87% specificity (Table 2). The majority of studies involved adults with haematological malignancies or undergoing stem cell transplantation; however, some studies also looked at patients undergoing solid organ transplantation and two dealt with paediatric patients. There were too few studies to carry out subgroup analysis though. Studies published since then have typically demonstrated the continued variation in reported performance with some studies on achieving sensitivities as low as 50% [243] and others 100% [254], with most studies in between [275, 293, 303]. Some studies in paediatric patients have demonstrated a complete lack of sensitivity of PCR [304] whilst in others sensitivities are similar to those seen in adult patients [291, 305]. This continued variation probably reflects the delays in the ability to adopt proposed standardised methods together with the realisation of the effects of concomitant antifungal therapy in patients who are tested (see below). Sun et al. [306] have published a meta-analysis of the use of BAL material for the PCR diagnosis of IA. They reviewed 17 studies that fitted the inclusion criteria between 1993 and 2009, including several pre-EORTC criteria studies where the criteria used for the definition of IA were deemed similar. Most studies focussed on patients with haematological malignancy and stem cell transplantation though a number of patients with solid tumours were also included. Overall sensitivity was high at 91% in proven and probable IA, with specificity 92% and a diagnostic odds ratio of 122. The authors commented on the variations in methodology and noted that commercial extraction systems appear to improve specificity though not sensitivity. Avni et al. [10] more recently have also published a meta-analysis including several more recent studies though this has not changed the sensitivity, and the overall specificity was slightly increased at 96%. These authors reviewed 10 studies where GM and PCR were directly compared on BAL samples. In seven studies where GM with a cutofff of 0.5 was compared to PCR, sensitivities and specificities (GM 82%, 97%; PCR 86% and 97%, resp.) were comparable. The results suggest combining both methods to maximise performance as has been advocated by others [89, 293, 307], an approach largely limited by cost. The GM assay unlike PCR is part of the EORTC criteria for defining probable cases of IA [308], and at-risk patients with relevant positive CT signs but negative GM (and other mycological results) are classified as possible IA. Bergeron et al. [38] have found that 66% of patients with possible IA in a recent study were PCR positive suggesting that if PCR-based assays can be standardised the assay can be incorporated into the EORTC criteria and many possible patients could be upgraded to probable disease leading to improved outcomes.

The effect of antifungal therapy on the sensitivity of PCR assays for IA has long been debated and McCulloch et al. 2012 suggest that their animal model shows evidence that antifungal therapy in an animal model reduces the rate of PCR detection [309]. Reinwald et al. 2012 systematically examined the effect of prior mould active antifungal therapy on the sensitivity of PCR on BALS specimens and demonstrated a decreased sensitivity where patients had received more than one agent prior to bronchoscopy [310]. This supports the findings from the AmBiLoad (high doses liposomal amphotericin B) trial where Hummel et al. 2010 attributed the low sensitivities of PCR testing to the use of AmBisome/AmBiLoad [311], and others have made similar observations in paediatric patients [312]. This contrasts with the findings of Musher et al. who found improved sensitivity for PCR detection of AI in BAL samples in patients on antifungal therapy compared to those who had not [313]. As these authors point out, this kind of analysis is problematic due to being subject to significant bias in terms of who is selected for antifungal therapy. In animal models where this bias can be overcome, treatment of IA reduces the sensitivity of all diagnostic tests reflecting the overall reduction of the burden of the fungus [103].

## 8. Other Biomarkers for IA

A recent test that has been developed and marketed for the detection of *Aspergillus *and diagnosis of IA is an antigen test detecting an extracellular glycoprotein antigen only produced during active growth of the fungus using a lateral flow device format [314]. The assay is specific for *Aspergillus* sp. and reacted positively to sera from patients diagnosed with IA by GM and BDG assay results [314, 315]. In a guinea pig model of IA, the assay flagged earlier than either GM or BDG [316]. Other *A. fumigatus* antigens that have been suggested as targets for a new assay include Cf2 on the surface of the growing fungus [317], other cell-wall associated antigens [318], proteinases [319] the proteins of the immunosecretome [320], and gliotoxin and its derivatives [321, 322].

Serum antibodies to *Aspergillus *have been suggested as a diagnostic approach for detection of IA; however, in the immunocompromised patient populations affected by this infection, antibody responses generally are poor [119]. Antibody assays may be useful in the case of IA seen in nonneutropenic patients [323, 324], or in the case of identifying patients at risk of IA prior to myeloablative chemotherapy, or stem cell transplantation [325]. Antibody responses to *Aspergillus *in patients with IA in a nonimmunocompromised background such as COPD have been discounted [48] but may in fact have a useful role (Datta et al., manuscript submitted).

Breath tests for pulmonary IA have been proposed for many years and a recent report of the successful detection of the 2-pentyl-furan in the breath of 2 patients with IA that resolved on treatment suggests that this novel approach may have provided evidence of proof of concept [326] and trials of this ultra-low interventional investigation are eagerly awaited.

## 9. Strategies 

Given an increasing number of diagnostic tools, many of which have been shown to perform well, the clinician managing a patient population at risk of IA needs to determine a strategy for when to intervene to provide best outcomes. A range of approaches to this problem have been formulated, whilst various terms using these approaches can be broadly categorised as prophylaxis, empiric, preemptive, and targeted (Table 4) [327]. 

Use of antifungal therapy with or without protective HEPA-filtered accommodation to prevent susceptible patients from succumbing to IA has been intensively researched and is controversial [328, 329]; however, most institutions will treat patients at risk of IA prophylactically. Effective prophylaxis will reduce reliance of laboratory diagnosis significantly and where this reduces pretest probabilities of IA in patient populations, the effectiveness of expensive laboratory tests is likely not to be cost-effective [330]. The emergence of resistance to azole antifungals, the mainstay of prophylaxis in *A. fumigatus*, in recent years [331], though not currently thought to result from prophylaxis, has led many to question the use of this approach. Empiric antifungal therapy has been the main approach in many centres for patients with haematological malignancies and undergoing stem cell transplantation, typically where patients are febrile and fail to respond to antibacterial therapy [332]. Laboratory investigations are either not carried out, or, if done, results are looked for to confirm the strategy and are possibly used to escalate or de-escalate the therapy. This strategy typically using amphotericin B is considered to improve survival [333], though there are very few trials against placebo and these only showed improvements in mortality attributed to invasive fungal infection and not overall mortality [334]. However, it has been estimated that 40%–50% of all neutropenic patients receive empiric antifungal therapy whilst only 5%–15% have infections [327]. Thus this is not an efficient approach, exposing many patients to unnecessary medication with concomitant risk [335] and the cost-effectiveness has been questioned [336]. It has also been suggested that fever is not only nonspecific but also may be a late marker for invasive fungal disease [337]. 

Thus some form of preemptive strategy utilising one or more specific markers for IA have been advocated by many for several years [335]. Retrospective studies of using HRCT to guide antifungal therapy in patients at risk from IA has shown to lead to earlier therapy [338, 339]. Serial CT scanning has been found in some studies to show signs of IA prior to serum GM [340]; however, for many centres there are likely to be logistic limitations to obtaining regular serial CT scans. Several strategies for the incorporation of rapid nonculture-based tests such as GM, BDG, and PCR together with HRCT investigations into management strategies have been proposed, stratified by current prophylaxis and risk of infection [337]. Individual studies of the effectiveness of these strategies include Maertens et al. [341] analysis of the use of daily GM screening combined with HRCT and bronchoscopy in neutropenic patients resulting in reduced antifungal use and earlier therapy in several patients. The importance of incorporating any strategy into a clinical or integrated care pathway which is systematic, multidisciplinary, and auditable has been argued [342]. Barnes et al. [343] incorporated GM and PCR into a neutropenic care pathway identifying 17 cases of probable or possible IA, reducing antifungals costs and with no excess mortality compared to previous years. Cuenca-Estrella looked at how to combine PCR and GM suggesting using two positive PCR test results together with GM for optimal performance [275]. They noted that positive PCR tests most often preceded GM positivity and HRCT signs. Girmenia et al. [344] described a pathway with the use of GM/CT-based investigations prompted by persistent febrile neutropenia finding 63% survival in patients with IA and reduced antifungal use. Millon et al. looked at applying PCR to GM positive specimens but found that this was often not useful in aiding the distinction between true and false GM positives but that overall combining PCR and GM testing to inform treatment decisions was the way forward [345].

The European Conference on Infections in Leukaemia (ECIL) published a review of biomarker use recommending diagnostic-driven strategy in haematological oncology patients with GM testing every 3-4 days together with clinical evaluations and HRCT [346]. While this publication accepted that there is moderate evidence for the use of BDG assay again in conjunction with other investigations, they stopped short of a recommendation of its incorporation into a routine diagnostic strategy citing issues that need resolving including the understanding of the value BDG screening compared to preemptive assays, the value of the assay in HRCT recipients and paediatric patients and in specimens other than sera, the integration of the assay with other markers, and the outcomes in patients where BDG is used in a diagnostic strategy. Perhaps surprisingly, the ECIL recommendations failed to endorse the use of PCR testing in haematological malignancy due to a lack of standardization [295]. A set of German guidelines on managing IA accepted that both empiric and preemptive strategies could be effective [347]. At a recent consensus conference on invasive fungal infections in 2010, it was suggested that while a diagnostic-driven strategy is what should be aimed for, the role of at least some screening assays in a diagnostic-driven approach may be unsuitable in patients on mould-active antifungal prophylaxis [348]. 

Targetted approaches to managing IA and other IFD either requiring evidence of proven disease through positive culture from a sterile site or histopathological evidence of tissue invasion, or probable disease defined by two or more positive signs or markers is in practice rarely used due to the risks of delay in starting therapy and the associated poorer response to therapy. 

The gold standard of analysis in clinical research is the random controlled trial (RCT) and there are no theoretical reasons why different diagnostic strategies cannot be subject to the same rigorous comparisons [349] except for the high cost and logistic difficulty. However, some RCTs of diagnostic approaches for IA or invasive fungal infections in at-risk patients have been reported. In an attempt to utilise the potential of the enhanced sensitivity and high positive predictive value of testing of PCR, Hebart et al. [350] randomised a cohort of allogeneic stem cell transplant patients to receive one-arm antifungal therapy after a single positive pan-fungal PCR or had febrile neutropenia unresponsive to antibacterial therapy or had in the other arm simple empiric therapy. The group with PCR-directed therapy received more antifungal therapy and showed better survival after 30 days (1.5% versus 6.3%) but not after 100 days. Eight cases of proven IA were seen in the PCR group and 5 in the control in both groups. Overall, the findings were perhaps disappointing but the results did suggest that a higher frequency of PCR screening might detect invasive fungal infections earlier than an empiric approach.

In a similar study of reduced intensity, conditioning allogenic stem cell transplantation patients were screened with an *Aspergillus* specific PCR test and patients with a single positive result were randomised for antifungal therapy with liposomal amphotericin or no intervention though all febrile neutropenic patients received antifungals on an empiric basis [351]. Interestingly in this study a single positive PCR result was found not to be associated with invasive fungal infection within the first 100 days after transplant; however, the value of PCR was in part not possible to determine due to the low number of cases of infection during this period. Furthermore, over half the patients with a single positive PCR result were not randomised due to clinicians rebelling against the use of intravenous antifungals in otherwise well patients. In all 20 of the cases of these patients with a single positive PCR result, the test failed to be reproducible. An alternative study design was adopted by Cordonnier et al. [352] who randomised a group of patients with haematological malignancies or autologous stem cell transplant recipients to receive either standard empiric antifungal therapy in the case of antibiotic resistant febrile neutropenia or to a preemptive therapy protocol. In the preemptive group, empiric therapy was withheld unless there was clinical or imaging evidence of fungal infection or a positive GM result, though a higher cutoff of 1.5 was being used at this time. There was no difference in survival between the groups though there were significantly more invasive fungal infections in the preemptive group (9.1% compared to 2.7%), and most of these were IA. Antifungal costs in the preemptive group were reduced by 35%. This study had several limitations such as the relatively high GM cutoff used, the low frequency use of this assay, and the omission of some high-risk patients [335]. However, this study has encouraged others to plan further RCTs with the adoption of diagnostic tests with high negative predictive values or logistic ratios in order to rule out fungal infections including IA where results are negative in patients being screened [353]. A mixed treatment analysis of empiric therapy compared to two of the above preemptive therapy approaches found no difference in all-cause mortality [354].

## 10. Using Laboratory Tests to Predict Outcomes

Efforts to improve the performance of diagnostic tests frequently focus on comparative studies and devising increasingly detailed “gold standard” case definitions. However, this leads to tests simply defining patients who fit into arbitrary diagnostic criteria and there has been a move in more recent years to understand the relationship between diagnostic tests for IA with clinical outcomes enabling clinicians not just to diagnose but also to predict outcomes to antifungal therapy and use the tests to monitor response. This linking of diagnostic test results to the outcome of therapy may also allow the tests to stand as surrogate markers in antifungal drug trials [355]. Simple correlations of the dynamics of serum GM and positive patients becoming negative with clinical outcome survival indicate a good correlation in individual studies [356–358] confirmed in a recent literature review [359]. In a more sophisticated analysis, Koo et al. [360] looked quantitatively at serum GM dynamics earlier in the disease process with outcomes in patients on antifungal therapy. They found a correlation between baseline GM levels and survival at 6 weeks. They were also able to quantify what many had observed anecdotally or in earlier studies [361, 362] that in patients with proven or probably IA for each unit rise in GM index with a 7-day period following diagnosis increased the risk of 6-week mortality by 25% and a decline of 1 unit reduced this risk by 22%. A similar exercise to this examining GM trajectories in patients compared to general clinical response and 12-week mortality also showed a correlation between GM decline and outcome in terms of clinical response, though not in terms of survival in patients with a positive (≥0.5) baseline GM result. However, in patients with a negative (<0.5) baseline GM, there was a clear correlation between increasing GM levels and both clinical response and survival at week 12 [363]. The authors used ROC analysis to demonstrate that any patient showing with a baseline GM of <0.5 and showing an increase of >0.13 within 2 weeks of baseline measurements increased the likelihood of mortality by week 12 by 2.4-fold. Bergeron et al. [358] did not find a correlation of changes in GM level in the first 10 days after a positive sample with outcome, but did see an overall correlation between baseline GM levels and 9-week survival. 

Nouer et al. have proposed incorporating GM dynamics into a new set of response criteria in patients diagnosed by a positive GM, where success is defined by the GM assay is becoming negative and remains negative for two or more weeks and failure by the persistent presence of GM in serum [11] GM does appear to be more a highly responsive marker for IA and was shown to decline to negative levels in 87% of patients who responded according to these criteria within 3 weeks. Combining analysis of GM normalisation with baseline neutrophil levels and creatine clearance may enhance the prediction of outcomes in IA and provide a simple way to optimise management. In the earliest studies of molecular diagnosis of IA, persistent positive PCR results were more likely to be seen in patients who did not survive, whilst patients whose blood became negative had better outcomes [239]. Furthermore, this same group [364] showed that in prior to undergoing bone marrow transplantation, patients with BAL specimens positive by PCR for *Aspergillus *were predictive of those developing IA during transplantation. Bergeron et al. [358] did not find any difference in outcomes in patients testing positive by a PCR assay for *Aspergillus *compared to those who did not. But in IA patients serially tested for *Aspergillus *RNA by NASBA, those dying whose death was attributed to IA were more likely to have became positive or remained positive for the NASBA test [365]. An analysis of changes in BDG levels in patients with a range of IFIs including IA found that short-term BDG increases did not correlate with survival and that BDG does decline but over a longer term which is of less practical clinical use [366]. Examination of the dynamics of general markers of inflammation such as C-reactive protein and interleukin 6 showed that a failure of these markers to decline after initiation of antifungal therapy was predictive of a poor response [65].

## 11. Conclusions

A vast effort has been expended and continues to be expended on the aim of being able to identify patients who have IA and who will benefit from some form of therapy. This reflects the continued disproportionate mortality associated with this disease despite major improvements in antifungal therapy together with a desire to manage patients at risk of IA more efficiently and with lower costs by targeting therapies [330]. 

Immunocompromised patient populations at risk, particularly those with low neutrophil counts, namely, those with haematological malignancies and undergoing stem cell transplantation, have been recognised for decades whilst there is also considerable understanding in other groups such as those with solid organ transplantation. Increasingly there is interest in risk of IA and methods of diagnosis in immunocompetent patient groups such as those with chronic lung disease which may require a very different approach [367].

Of the wide variety of methods reviewed of diagnosing IA, direct microscopy and histopathology are undoubtedly the most subjective, skilled, and labour intensive. Though histopathological detection of *Aspergillus* of tissue will remain an essential capacity and help define the highest level of certainty in diagnostic criteria [368], less invasive and more rapid methods have already overtaken this approach in most settings. Some will also consider that traditional culture-based methods may also be superceded by DNA and antigen detection methods. However, in the recent US PATH registry update culture of *Aspergillus *for the diagnosis of IA was still the most frequent laboratory approach [369]. Furthermore, cultures of *Aspergillus *allow further analysis such as susceptibility to testing, recent developments in direct molecular detection of resistance notwithstanding, and the possibility of molecular typing in epidemiological investigations [370]. Furthermore, new species of *Aspergillus* causing invasive disease continue to be uncovered by culture whose applicability to serological and molecular detection cannot be assumed [27]. Culture of respiratory specimens in clinical laboratories is unlikely to be abandoned in the near future and culture of *Aspergillus* will remain an important if generally insensitive approach to the diagnosis of IA. 

Chest HRCT scanning for the presumptive diagnosis of pulmonary IA has revolutionised approaches to this disease in recent decades and become the third pillar of the probable category of IA. However, CT signs still lack specificity and it remains to be seen if developments in methods to provide a more specific identification of lesions as relating to *Aspergillus* will lead to a second revolution and the reduction in need to send specimens to the pathology laboratory. 

The detection of galactomannan in clinical specimens is firmly established as the test of choice for any laboratory providing diagnostic services for patients at risk of IA. Serum detection remains a key approach and amenable to serial measurements whilst BAL material appears to have a higher sensitivity but is often harder to obtain. False positives are less of an issue than is often claimed with antibiotic sources of GM now rarely seen and neonatal samples likely to account for a tiny fraction of samples analysed. Sensitivity is clearly affected by antifungal treatment, though the biological basis for this is unclear, this remains the main limitation to the use of the assay particularly in patients undergoing antifungal prophylaxis. A plethora of meta-analyses have confirmed the clinical value of GM testing and at least some analyses of GM serum kinetics point to the potential for this assay to guide therapy beyond diagnosis. The BDG assay has yet to be widely adopted as a diagnostic tool, perhaps suffering from the need to also require more specific diagnostic methods to target therapy in positive patients. Meta-analysis suggests that problems with specificity were probably overestimated in initial studies and sensitivity is comparable with GM. The BDG assay will probably find its place in some centres in carefully designed care pathways where the negative predictive value can be used to reduce empiric therapy. The PCR assay to detect *Aspergillus *DNA held a large amount of promise but has been limited up until recently by a lack of standardisation. The scientists of EAPCRI have nobly met the challenge of technical diversity in PCR assays and provided meticulously researched standard methods for extraction of DNA from whole blood and serum. The continuing introduction of commercial PCR assays will consolidate this standardisation. These advances are likely to lead to easier comparisons with other methods and incorporation of this form of test into the EORTC criteria for research use. Preemptive therapy is based on assessing risk in patients as fully as possible, and increasingly underlying aspects of disease and routine cell and biochemical markers are being found to be relevent to this assessment. In a recent fascinating examination of patients with IA it was shown that patients with allogeneic SCT were more likely to exhibit airways rather than angio-invasive disease (as shown by HRCT) and higher leukocyte counts and were more likely to provide a positive culture yield on bronchoscopy compared to patients with acute leukaemia [38]. How to decide on the best blood-based assay to use and in what way has exercised the minds of haematologists and clinical microbiologists for some time. This is an area where opinions are divided. It is difficult to reconcile statements such as “highly sensitive and specific methods for the detection of an early fungal infection are yet unavailable” made in 2012 [371] with significant successes of others using a preemptive approaches using antigen and PCR detection [343]. It is generally agreed that an RCT of empiric compared to preemptive diagnostic-driven therapy, probably comparing two or more laboratory tests [353], is the way forward, though the few undertaken so far have had significant limitations. Ensuring rapid diagnosis as delays may impact on long-term outcomes [61]. To the authors knowledge, currently two more such studies are underway an Australian study which was started in 2005 but has still to report (NCT00163722) an EORTC sponsored study started in 2012 (NCT01288378). Others have made the case to compare preemptive approach with a patient population treated with antifungal prophylaxis [335]. 

New assays continue to be described and many will doubtlessly fall at early hurdles due to poor performance or lack of investment whilst competing against many suboptimal but well-established competitors. Predicting the future of developments in the diagnosis of IA is probably harder than predicting outcomes in the disease itself, but this author is particularly interested to see positive developments in the minimally invasive breath tests and RNA detection with its promise of detecting actively growing as compared to colonising *Aspergillus*. 

People with fewer than the normal level of neutrophils continue to die of IA despite all the developments in diagnosis and treatment. It a cliché but also the truth to say that the challenge continues and the improvements in outcomes seen in the last decades can be built upon as we continue to understand the complex interaction between the fungi of the genus *Aspergillus* and humans with damaged host defences.

## Figures and Tables

**Table 1:** Antifungal susceptibilities for different species of *Aspergillus*.

Species	Resistance to	Frequency
*A. fumigatus *	Itraconazole	Occasional, increasing
	Voriconazole	Rare, increasing?
	Posaconazole	Rare, increasing?
	Echinocandins	Rare
*A. lentulus *	Amphotericin B	Intrinsic?
	Itraconazole	Intrinsic?
	Voriconazole	Intrinsic?
*A. fumigatiaffinis *	Amphotericin B	Intrinsic?
	Itraconazole	Intrinsic?
	Voriconazole	Intrinsic?
*A. terreus *	Amphotericin B	Intrinsic
*A. flavus *	Amphotericin B	rare
	Itraconazole	rare
	Voriconazole	rare
*A. niger *	Itraconazole	Occasional
*A. awamori *	Itraconazole	Occasional
*A. tubingensis *	Itraconazole	Intrinsic?
*A. acidus *	Itraconazole	Intrinsic?

**Table 2:** Main approaches to laboratory diagnosis.

Test	Specimens	Advantages	Disadvantages
Direct microscopy	Respiratory	Low cost	Insensitive, labour intensive
Culture	Respiratory, tissue	Low cost, enables further analysis	Insensitive
Histopathology	Tissue	Enables proven diagnosis	Requires biopsy tissue
Galactomannan (GM)	Serum, BAL	Sensitive, specimens easy to obtain	Lacks sensitivity in patients on antifungals
*β*-D-glucan (BDG)	Serum	Sensitive, specimens easy to obtain	Lacks specificity
PCR (DNA detection)	Any	Sensitive, can be applied to any specimen	Labour intensive, expensive

**Table 3:** Diagnostic accuracy of the main laboratory markers for IA.

Reference	Method	Sample	Sensitivity^a^	Specificity^a^	DOR^b^
Mengoli et al. 2009 [183]	PCR (1)	Blood	0.88 (0.75–0.94)	0.75 (0.63–0.84)	22.11 (7.77–62.92)
Mengoli et al. 2009 [183]	PCR (2)	Blood	0.75 (0.54–0.88)	0.87 (0.79–0.93)	21.33 (6.86–46.63)
Leeflang et al. 2008 [180]	GM	Blood	0.79 (0.61–0.93)	0.82 (0.71–0.83)	17.10^c^
Sun et al. 2010 [182]	GM	Blood	0.66 (0.61–0.70)	0.9 (0.89–0.90)	19.1 (12.67–28.79)
Guo et al. 2010 [184]	GM	BAL	0.86 (0.70–0.94)	0.89 (0.85–0.92)	51.0^c^
Onishi et al. 2012 [185]	BDG	Blood	0.77 (0.71–0.82)	0.83 (0.82–0.84)	23.2 (9.9–54.4)

^a^Sensitivity and specificity as proportions with 95% confidence intervals (CI) given in brackets, ^b^DOR diagnostic odds ratio, ^c^calculated from sensitivity and specificity data in the references, 95% CI not calculable.

**Table 4:** Approaches to the prevention and diagnosis of IA.

Strategy	Rationale	Laboratory diagnostic use
Prophylaxis	Prevention of infection using antifungal treatment and protective accommodation	None
Empiric	Early antifungal therapy in response to non-specific signs and symptoms	None (blood cultures are typically done to detect bacteremia and fungemia)
Preemptive	Antifungal therapy in response to early specific markers of IA	CT, GM, PCR, and BDG usually one or two markers triggering therapy
Targetted	Antifungal therapy of clearly defined cases of IA	All available investigations, using EORTC-MSG criteria
